# Multicentric Study on the Clinical Mycology Capacity and Access to Antifungal Treatment in Portugal

**DOI:** 10.1007/s11046-024-00830-9

**Published:** 2024-01-24

**Authors:** Raquel Fernandes, Raquel Sabino, Cristina Cunha, Oliver A. Cornely, Agostinho Carvalho, Jon Salmanton-García, PT Group for IFI Management Capacity, Joana Batista, Dinah Carvalho, Adriana Coutinho, Flávia Cunha, Augusta Gonçalves, Catarina Gouveia, António Guerra Maio, Augusto Machado e Costa, Dolores Pinheiro, Pedro Póvoa, Elmano Ramalheira, Valentina Santos, Ana Cristina Silva, Zélia Videira

**Affiliations:** 1 Life and Health Sciences Research Institute (ICVS), School of Medicine, University of Minho, Campus de Gualtar, 4710-057 Braga, Portugal; 2 ICVS/3B’s - PT Government Associate Laboratory, Braga, Guimarães, Portugal; 3 Reference Unit for Parasitic and Fungal Infections, Department of Infectious Diseases, National Institute of Health Doutor Ricardo Jorge (INSA), Lisbon, Portugal; 4 Faculdade de Medicina, Instituto de Saúde Ambiental, Universidade de Lisboa, Lisbon, Portugal; 5 Laboratório Associado TERRA—Laboratório para o Uso Sustentável da Terra e dos Serviços dos Ecossistemas, Instituto Superior de Agronomia, Lisbon, Portugal; 6 Faculty of Medicine and University Hospital Cologne, Institute of Translational Research, Cologne Excellence Cluster On Cellular Stress Responses in Aging-Associated Diseases (CECAD), University of Cologne, Herderstr. 52, 50931 Cologne, Germany; 7 Faculty of Medicine and University Hospital Cologne, Department I of Internal Medicine, Center for Integrated Oncology Aachen Bonn Cologne Duesseldorf (CIO ABCD) and Excellence Center for Medical Mycology (ECMM), University of Cologne, Cologne, Germany; 8 German Centre for Infection Research (DZIF), Partner Site Bonn-Cologne, Cologne, Germany; 9 Faculty of Medicine and University Hospital Cologne, Clinical Trials Centre Cologne (ZKS Köln), University of Cologne, Cologne, Germany

**Keywords:** Portugal, Clinical mycology, Invasive fungal disease, Diagnosis, Therapy, Antifungals

## Abstract

The success of the clinical management of invasive fungal diseases (IFD) is highly dependent on suitable tools for timely and accurate diagnosis for effective treatment. An in-depth analysis of the ability of European institutions to promptly and accurately diagnose IFD was previously conducted to identify limitations and aspects to improve. Here, we evaluated and discussed the specific case of Portugal, for which, to our knowledge, there are no reports describing the national mycological diagnostic capacity and access to antifungal treatment. Data from 16 Portuguese medical institutions were collected via an online electronic case report form covering different parameters, including institution profile, self-perceived IFD incidence, target patients, diagnostic methods and reagents, and available antifungals. The majority of participating institutions (69%) reported a low-very low incidence of IFD, with *Candida* spp. indicated as the most relevant fungal pathogen, followed by *Aspergillus* spp. and *Cryptococcus* spp. All institutions had access to culture and microscopy, whereas 94 and 88% were able to run antigen-detection assays and molecular tests, respectively. All of the institutions capable of providing antifungal therapy declared to have access to at least one antifungal. However, echinocandins were only available at 85% of the sites. Therapeutic drug monitoring (TDM) was reported to remain a very restricted practice in Portugal, being available in 19% of the institutions, with the TDM of itraconazole and posaconazole performed in only 6% of them. Importantly, several of these resources are outsourced to external entities. Except for TDM, Portugal appears to be well-prepared concerning the overall capacity to diagnose and treat IFD. Future efforts should focus on promoting the widespread availability of TDM and improved access to multiple classes of antifungals, to further improve patient outcomes.

## Introduction

Invasive fungal diseases (IFD) are a global burden affecting more than 150 million individuals worldwide and lead to more than 1.7 million deaths every year [1–3]. Furthermore, IFD are associated with a significant socioeconomic burden, due to the elevated number of hospitalizations and the extreme healthcare costs, estimated to surpass $7 billion annually in the US [4], with similar figures in Europe [5]. With the advent of modern medicine and advances in medical care, the number of susceptible hosts, namely hematological and oncological patients, recipients of hematopoietic stem-cell and solid organ transplantation, and immunosuppressed patients receiving chronic steroids and biologic therapies, is increasing [6–8]. Other vulnerable settings include advanced age and intensive care, and severe debilitating conditions, namely respiratory viral infections, such as COVID-19 and influenza, which underlie the development of COVID-19-associated aspergillosis (CAPA) [9, 10] and COVID-19-associated mucormycosis [11], and influenza-associated aspergillosis (IAPA) [12], respectively. Traveling to endemic regions further potentiates the dissemination of endemic mycoses to non-endemic areas [13–15], which in the case of Portugal, may be the reflection of the high number of individuals of South American and African origin [14].

Diagnosing IFD poses a significant challenge. Not only are available tools limited, but affected patients typically present non-specific symptoms or have underlying conditions masking the disease [16]. Individuals with chronic obstructive pulmonary disease (COPD), or bronchiectasis, for instance, not only experience respiratory symptoms that overlap with those of fungal infections but also exhibit challenging radiological presentations since they may be difficult to distinguish from the inherent clinical presentations of the underlying condition itself [17]. In some instances, such as the diagnosis of *Candida* infections, the correct distinction between colonization and active infection is also difficult. Early diagnosis, however, can improve treatment outcomes and potentially alleviate the financial burden associated with IFD [18].

A major determinant dictating the accessibility to these techniques has been reported to lie in the economic status of the country, with a previous study on the diagnostic capacity of European institutions describing considerable differences among countries according to their gross domestic product (GDP) [19]. On the other hand, in some cases, such as Italy [20] and Argentina [21], the resources seemed to be homogeneously distributed with no significant differences among the evaluated national institutions.

Portugal, a Western European country whose estimated population is approximately 10.3 million inhabitants [22], was categorized as an European median-income country in our previous nationwide censoring survey, with a GDP encompassing the US$30,000–$45,000 range [19]. The Portuguese healthcare system includes both public and private medical care providers. The public healthcare system, known as the National Healthcare System (NHS), is a free healthcare service including all the public entities designated for the provision of medical care services [23]. The NHS is a platform created to provide equitable treatment among patients regardless of their socioeconomic status, being financially supported mainly through taxation. This medical system has presented good performance outcomes. For example, rates of avoidable hospitalizations for asthma and COPD, conditions well-recognized for their established connection to an elevated risk of IFD development [24, 25], ranked amongst the top performers within the Organization for Economic Cooperation and Development [26].

Our study aimed to describe the diagnostic capacity of IFD and the global access to antifungal treatments in public Portuguese institutions and identify the most critical aspects for improvement.

## Methods

A comprehensive questionnaire-based approach was implemented at multiple centers between November 1, 2021, to May 31, 2023. The data was gathered using an electronic case report form hosted on the website www.clinicalsurveys.net/uc/IFI_management_capacity/ (EFS Summer 2021, TIVIAN GmbH, Cologne, Germany). Rigorous validation of responses was undertaken to ensure the accuracy, coherence, and completeness of the data.

The primary objective of the survey was to evaluate various critical aspects related to the diagnosis and treatment of IFD. These aspects encompassed the examination of institutional profiles, the assessment of perceptions regarding IFD incidence and significance at each institution, the exploration of microscopy techniques, culture, and fungal identification methods, analysis of serology approaches, antigen detection capabilities, molecular assays, and the availability of therapeutic drug monitoring (TDM). Participants were required to provide binary responses indicating the accessibility of specific methods at their respective locations. Additionally, for serology, antigen detection, molecular testing, and TDM, laboratories were asked to specify whether these services were available onsite or outsourced to external institutions.

The prevalence of IFD was estimated using a Likert scale, allowing participants to rate the incidence on a scale from 1 (extremely low) to 5 (very high) (Table 1). To ensure a diverse and representative group of participants, researchers reached out to individuals from various regions in Portugal through mass e-mails, targeting both close collaborators of the authors and members of key scientific organizations such as the International Society of Human and Animal Mycology (ISHAM; www.isham.org) and the European Confederation for Medical Mycology (ECMM; www.ecmm.info).

**Table 1:** Baseline characteristics of participating institutions in Portugal

Participating institutions	n	% (of replies)
	16	100.0
Patients at care		
COVID-19	12	75.0
Diabetes mellitus	14	87.5
Hematology	12	75.0
HIV/AIDS	12	75.0
Oncology	13	81.3
Parenteral nutrition	14	87.5
Solid organ transplantation	5	31.3
Stem cell transplantation	5	31.3
Invasive fungal infection incidence		
Very low	5	31.3
Low	6	37.5
Mild	3	18.8
High	2	12.5
Very High	0	0.0
Most relevant pathogen(s) within institutions		
*Aspergillus* spp.	12	75.0
*Candida* spp.	16	100.0
*Cryptococcus* spp.	5	31.3
*Fusarium* spp.	2	12.5
Mucorales	1	6.3

The collected data was analyzed and summarized using frequencies and percentages. For all statistical analyses, SPSS v27.0 (SPSS, IBM Corp., Chicago, IL, United States) was utilized.

## Results

**Fig. 1:**
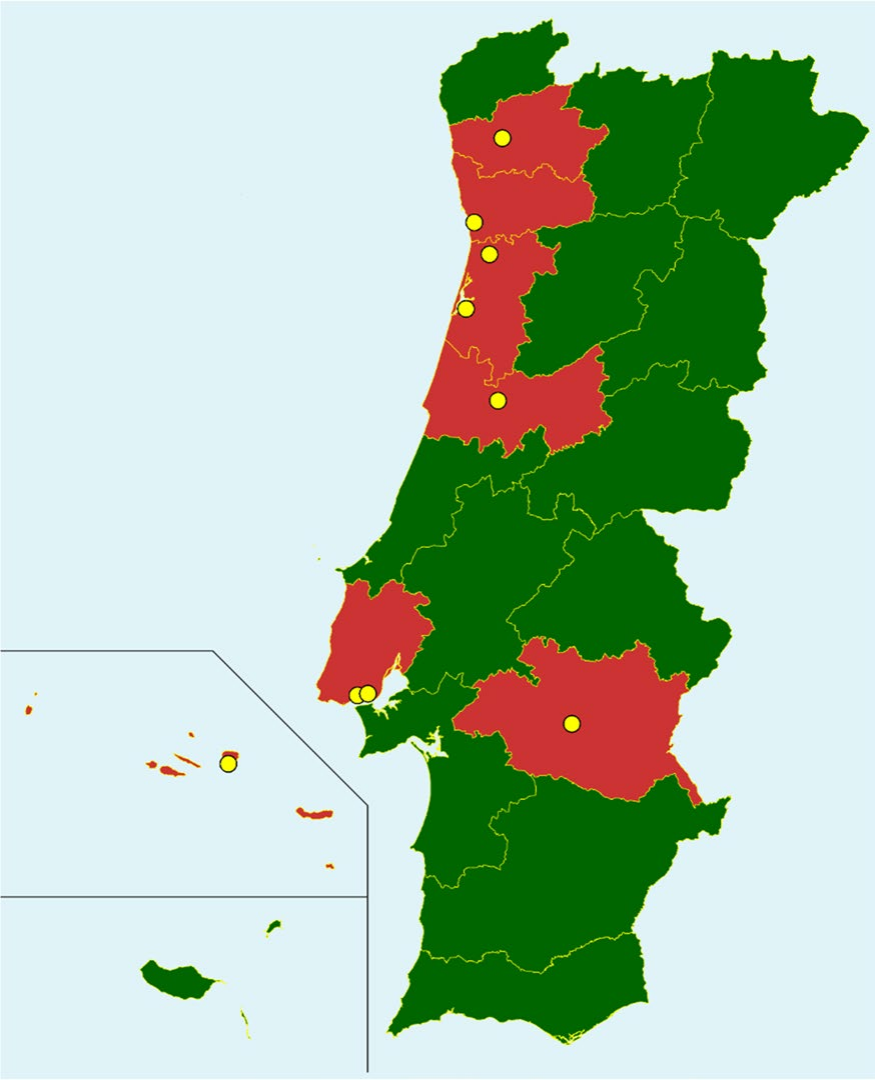
Map of participating institutions per district. Districts with participating institutions are colored in red. Districts whose centers have not been included are colored in green. If more than one participating center (in yellow) is from the same city, a single point is pictured

During the study period, a total of 16 Portuguese institutions self-assessed their capability to manage invasive fungal infections (Fig. 1).

Table 1 describes the baseline characteristics of the participating institutions in Portugal. Of the 16 participating institutions, 14 (87.5%) were admitting patients with diabetes mellitus or in need of parenteral nutrition, 13 (81.3%) patients with solid or hematological cancer, and 12 (75%) with COVID-19, human immunodeficiency virus (HIV), or blood or bone-marrow related disorders. Participants were inquired about their perception of IFD incidence within their respective institutions. Out of the 16 participating institutions, 11 (68.8%) reported a low-very low incidence of IFD. A high incidence of IFD was reported in 2 (12.5%) institutions, whereas a very high incidence was not perceived at any site. Participants also listed the most relevant pathogens at each institution, with *Candida* spp. (n = 16, 100%) being pointed out by all institutions as the most significant pathogen, followed by *Aspergillus* spp. (n = 12, 75%), *Cryptococcus* spp. (n = 5; 31.3%), *Fusarium* spp. (n = 2, 12.5%) and Mucorales (n = 1, 6.3%).

Microscopy techniques were available in all 16 (100%) institutions (Table 2). When cryptococcosis was suspected, microscopy was the method of choice to perform a direct examination of body fluids for most institutions (n = 14, 87.5%). However, only 3 (18.8%) employed direct microscopy or silver staining for suspected cases of mucormycosis and pneumocystosis, respectively. Of note, less than half of the participating institutions (n = 7, 43.8%) had access to fluorescent dyes. China or India ink were the most widely available staining dyes (n = 15, 93.8%), followed by Giemsa staining (n = 10, 62.5%), potassium hydroxide (n = 8, 50%), silver staining (n = 4, 25%), and calcofluor white staining (n = 1, 6.3%). Access to culture media was also widespread (100%), with all 16 institutions offering tests for specific identification. The most prevalent type of test was based on automated identification through VITEK 2® and other commercial tests (n = 16, 100%), followed by classical biochemical tests (n = 11, 68.8%), matrix-assisted laser desorption ionization time-of-flight mass spectrometry (MALDI-TOF MS) (n = 10, 62.5%), deoxyribonucleic acid (DNA) sequencing (n = 6, 37.5%) and macro and microscopic observation of the colonies (n = 5, 31.3%). Antifungal susceptibility testing was available in 13 (81.3%) institutions. The VITEK 2® system was the most popular technology employed in these settings (n = 10, 62.5%), followed by the E-test® (n = 9, 56.3%), and broth microdilution published by the European Committee on Antimicrobial Susceptibility Testing (EUCAST; n = 7, 43.8%) and Clinical and Laboratory Standards Institute (CLSI; n = 4, 25%). Remarkably, antifungal susceptibility tests were not available in 2 (12.5%) of the institutions. Molecular diagnosis is prevalent in Portugal with only 1 institution lacking this type of resource. Polymerase chain reaction (PCR) for *Pneumocystis* detection emerged as the most frequent among Portuguese institutions (n = 14, 87.5%), followed by *Aspergillus* (n = 12, 75%), *Candida* (n = 11, 68.8%), and Mucorales (n = 8, 50%). Of note, outsourcing of antibody and antigen detection and molecular techniques to external entities is a highly frequent practice, often prevailing over onsite procedures.

**Table 2:** Available methodologies for the phenotypic and molecular identification of fungal species and diagnosis of IFD in Portugal

Participating institutions	n	% (of replies)
	16	100.0
Microscopy	16	100.0
Available microscopy methods		
Calcofluor white	1	6.3
Giemsa stain	10	62.5
China/India ink	15	93.8
Potassium hydroxide	8	50.0
Silver stain	4	25.0
Fluorescence dyes	7	43.8
Direct examination of body fluids when cryptococcosis suspicion	14	87.5
Silver staining in pneumocystosis suspicion	3	18.8
Direct microscopy when mucormycosis suspicion	3	18.8
Culture and fungal identification	16	100.0
Fungal culture methods		
Agar Niger	1	6.3
Chromogen	2	12.5
Lactrimel agar	1	6.3
Potato dextrose agar	2	12.5
Sabouraud dextrose agar	12	75.0
Sabouraud dextrose agar + chloramphenicol	8	50.0
Sabouraud dextrose agar + gentamicin	5	31.3
Selective agar (chloramphenicol + cycloheximide)	6	37.5
Available tests for species identification		
Automated identification (VITEK, other commercial tests)	16	100.0
Biochemical tests	11	68.8
DNA sequencing	6	37.5
MALDI-TOF	10	62.5
Mounting medium	5	31.3
Available methods for determination of antifungal susceptibility profile		
CLSI (broth microdilution)	4	25.0
EUCAST (broth microdilution)	7	43.8
E-test® (gradient agar diffusion)	9	56.3
VITEK2®	10	62.5
Molecular diagnosis (PCR) †	15	93.8
*Aspergillus* spp.	12	75.0
Onsite	4	33.3
Outsourced	8	66.7
*Candida* spp.	11	68.8
Onsite	4	36.4
Outsourced	7	63.6
*Pneumocystis* spp.	14	87.5
Onsite	8	57.1
Outsourced	6	42.9
Mucorales spp.	8	50.0
Onsite	2	25.0
Outsourced	6	75.0

^†^The percentages of on site or outsourced molecular diagnostics (PCR) were calculated with regard to the total number of institutions that had access to each of those methodologies

Serological antibody detection was available in 14 (87.5%) institutions, of which 12 had the capacity to detect *Aspergillus* spp. (75%) antibodies (Table 3). In contrast, less than half of the institutions were equipped to detect antibodies against *Candida* spp. and *Histoplasma* spp. (n = 7, 43.8%). Antigen-detection assays were available in nearly all institutions (n = 15, 93.8%) except for one. *Aspergillus* and *Cryptococcus* antigen assays were available in 12 institutions (75%), whereas *Candida* and *Histoplasma* assays were limited to only 7 (43.8%) and 6 (37.5%), respectively. Tests to detect galactomannan were available in half of the institutions. Among these, 5 (31.3%) resorted to enzyme-linked immunosorbent assay (ELISA), and 6 (37.5%) to both lateral flow assay (LFA) and lateral flow device (LFD). Regarding *Cryptococcus*, the most commonly used detection assay was the latex agglutination assay (n = 9, 56.3%), followed by LFA (n = 6, 37.5%). Beta-glucan was also targeted for detection in 7 (43.8%) institutions.

**Table 3:** Available methodologies for fungal species serological detection and identification and diagnosis of IFD in Portugal

Participating institutions	N	% (of replies)
	16	100.0
Antibody detection †	14	87.5
*Aspergillus* spp.	12	75.0
Onsite	6	50.0
Outsourced	6	50.0
*Candida* spp.	7	43.8
Onsite	1	14.3
Outsourced	6	85.7
*Histoplasma* spp.	7	43.8
Onsite	3	42.9
Outsourced	4	57.1
Antigen detection †	15	93.8
*Aspergillus* spp.	12	75.0
*Aspergillus* spp. galactomannan (ELISA)	10	62.5
Onsite	5	50.0
Outsourced	5	50.0
*Aspergillus* spp. galactomannan (LFA)	6	37.5
Onsite	2	33.3
Outsourced	4	66.7
*Aspergillus* spp. galactomannan (LFD)	6	37.5
Onsite	1	16.7
Outsourced	5	83.3
*Candida* spp.	7	43.8
Onsite	1	14.3
Outsourced	6	85.7
*Cryptococcus* spp.	12	75.0
*Cryptococcus* spp. (LAT)	9	56.3
Onsite	9	100
Outsourced	0	0
*Cryptococcus* spp. (LFA)	6	37.5
Onsite	4	66.7
Outsourced	2	33.3
*Histoplasma* spp.	6	37.5
Onsite	2	33.3
Outsourced	4	66.7
β-glucan	7	43.8
Onsite	2	28.6
Outsourced	5	71.4

^†^ The percentages of onsite or outsourced antibody or antigen detection tests were calculated concerning the total number of institutions that had access to each of those methodologies

All 13 institutions that operate as treating centers and are capable of providing antifungal therapy had access to at least one antifungal (Table 4). The most frequently available antifungal agents in Portuguese institutions belonged to the triazole, polyene (n = 13, 100.0%), and echinocandin (n = 11, 84.6%) classes. Among these classes, the most common drugs available included liposomal amphotericin B (n = 11, 84.6%) within the polyene formulations, voriconazole and fluconazole (n = 13, 100.0%) within the triazoles, and micafungin (n = 11, 84.6%) within the echinocandins. The availability of other drugs, namely the allylamine terbinafine (n = 7, 53.8%) or the pyrimidine analog flucytosine (n = 5, 38.5%), was less frequent. According to the obtained data, TDM remains a very limited practice in Portugal, with only 3 institutions (18.8%) engaging in this type of patient follow-up. Three (18.8%) institutions were able to monitor patients receiving voriconazole, whereas only 2 (12.5%) and 1 (6.3%) sites, respectively, had flucytosine, and itraconazole and posaconazole monitoring available. However, this monitoring was conducted mainly through outsourcing, with only 1 institution performing flucytosine and voriconazole onsite.

**Table 4:** Available antifungal drugs and therapeutic monitoring for clinical management in Portuguese institutions

Participating institutions	n	% (of replies)
	16	100.0
Available antifungals	13	100.0
Amphotericin B	13	100.0
Amphotericin B deoxycholate	5	38.5
Amphotericin B lipid complex	4	30.8
Amphotericin B liposomal	11	84.6
Echinocandins	11	84.6
Anidulafungin	5	38.5
Caspofungin	8	61.5
Micafungin	11	84.6
Triazoles	13	100.0
Fluconazole	13	100.0
Isavuconazole	10	76.9
Itraconazole	7	53.8
Posaconazole	9	69.2
Voriconazole	13	100.0
Flucytosine (5-FC)	5	38.5
Terbinafine	7	53.8
Therapeutic drug monitoring †	3	18.8
Flucytosine (5-FC)	2	12.5
Onsite	1	50.0
Outsourced	1	50.0
Itraconazole	1	6.3
Onsite	0	0.0
Outsourced	1	100.0
Posaconazole	1	6.3
Onsite	0	0.0
Outsourced	1	100.0
Voriconazole	3	18.8
Onsite	1	33.3
Outsourced	2	66.7

^†^The percentages of onsite or outsourced TDM were calculated concerning the total number of institutions that had access to each of those methodologies

## Discussion

We conducted a systematic analysis of the capacity of Portuguese institutions to diagnose and treat IFD. Overall, over half of the participating institutions documented a low IFD incidence. However, it is important to note the subjective nature of the reported perceptions. *Candida* spp. was identified as the most prominent pathogen, followed by *Aspergillus* spp. and *Cryptococcus* spp., which collectively occupy the top positions on the World Health Organization (WHO) fungal priority pathogen list [27]. All institutions were prepared for fungal identification using culture and microscopy techniques, with the majority also endowed with antibody detection by serology, molecular tests, and antigen-detection assays. Antifungal drugs were generally accessible, with triazoles and amphotericin B being more widely available than echinocandins. TDM, however, stood out as one of the most important limitations, showing very restricted implementation.

Portugal follows the same pathogen prevalence pattern observed in European settings [19]. Notably, while other European countries, such as Italy [20] and Austria [28], identified *Aspergillus* spp. as their most important pathogen, Portugal aligns with the overall trends in Asian [29] and African [30] countries, where *Candida* spp. stands out as the predominant fungal pathogen. This mirrors older reports on the epidemiology of IFD in which *Candida* species were the main etiological agent [31]. However, the epidemiological landscape has markedly changed, with not only an increase in the prevalence of *Aspergillus* species but also the emergence of rare fungi, such as *Fusarium* and Mucorales [32].

The relevance given to *Aspergillus* spp. also follows a previous report from a Portuguese multicentric surveillance program, in which *Aspergillus* spp. was identified as the most predominant etiological agent of IFD [14]. This change in epidemiology is, at least in part, aligned with the widespread introduction of broad-spectrum antifungal prophylaxis. These preventive measures have been adopted to circumvent the challenges associated with diagnosis. Nonetheless, they often lead to the positive selection of resistant strains, possibly contributing to the emergence of difficult-to-treat fungi*.*

Although Portugal follows the same overall epidemiological pattern observed in Europe, *Cryptococcus* spp. exhibited a higher prevalence. A previous study had already established the higher incidence of cryptococcal meningitis among human immunodeficiency virus (HIV)-infected patients in Portugal compared to other European countries [15, 33–35], a difference that might be attributed to the high number of patients without antiretroviral treatment.

In contrast to the European landscape, and despite being less frequent, *Fusarium* was more prevalent in the Portuguese territory than fungi from the Mucorales order. Indeed, the incidence of fusariosis in Portugal has been increasing since the 90s. Although this survey did not include data from Madeira Island, previous reports state the introduction and settling of *Fusarium* in this geographical area [36]. Importantly, the geoclimatic conditions of Madeira can be suitable for the development and dispersion of these plant-pathogenic fungi [37, 38]. However, no genotyping studies are available to allow the comparison of the involved fungal strains and confirm this hypothesis.

Despite Portugal is not considered an endemic area for dimorphic fungi, the Portuguese multicentric surveillance program demonstrated a relatively high frequency of infections caused by these pathogens [14]. Case reports have documented the presence of IFD established by other endemic fungi such as *Paracoccidioides brasiliensis* and *Histoplasma* spp. [14, 15], which could be explained by the significant influx of immigrants from Africa and Brazil [39]. While our study did not report these endemic fungi, not all eligible institutions were surveyed and there is also the possibility of misdiagnosis or underdiagnosis at the participating centers. With this in mind, it is crucial to consider endemic mycoses in the clinical diagnosis of immunocompromised patients who were born in or traveled to endemic areas.

Direct microscopy is the method of choice in Portugal and Europe for fungal identification [19]. Among the stainings used, China/India ink and Giemsa are the most commonly accessible. Despite China/India ink being mostly used for *Cryptococcus* detection and associated with low sensitivity, its accessible price puts it on top of the available stains [40, 41]. The popularity of Giemsa stain might be explained not only by its efficient staining but also by its cost-effectiveness. Conversely, the fluorescent dye calcofluor white is a very scarce resource across institutions due to its comparatively higher costs, compromising the identification of *Aspergillus* and Mucorales, for which it is strongly recommended [42–44]. This limitation is in line with what has been reported among European institutions, where countries with a GDP inferior to 45,000$, such as Portugal, had limited or no access to this fluorescent dye [19].

Besides microscopy, the identification of fungal species involved predominantly automated identification, biochemical tests, and mounting medium. Of note, while in countries with a GDP exceeding $45,000, advanced and expensive diagnostic techniques were generally more accessible, countries with lower GDPs relied more frequently on more cost-effective techniques, considered obsolete, and surpassed by superior methods in high-income countries. These observations illustrate how the effectiveness of IFD diagnosis is influenced by the economic power of a country and how patient outcomes are contingent upon this factor. Of interest, MALDI-TOF MS was the most commonly available resource after automated identification and biochemical tests, being the most effective platform for microorganism identification [45]. Strong performance and notable investment-to-return ratios have rendered Portugal well-prepared in the most recent Euro Health Consumer Index Report (2018), an annual classification of national healthcare systems in Europe [46]. This philosophy adopted by the NHS might explain the high availability of MALDI-TOF MS, despite its price.

Antibody and antigen detection assays are readily available, with over half of the institutions outsourcing these techniques. The exception is the detection of *Cryptococcus* spp. antigen, which is primarily conducted onsite. Molecular diagnosis is also often performed by external entities, although *Pneumocystis* spp. PCR is widely accessible and frequently performed onsite. It is important to note that while outsourcing widens the pool of resources available, it also comes with significant bureaucratic processes, including requests and approvals that can cause delays in responses, hindering timely clinical decisions. Hence, prioritizing the implementation of onsite resources should be a primary focus when aiming to improve the diagnosis and treatment of IFD.

The frequency of antifungal susceptibility testing in Portugal is lower than the overall European frequency [19]. Antifungal susceptibility testing is a resource in need of improvement, as its availability is not only below the European average but also the number of institutions conducting it onsite is limited. However, it is important to mention that Portugal is still ahead of other non-European countries, namely from the African continent [30], and Latin America and the Caribbean [47], which have significantly larger populations and face critical situations regarding fungal infections.

In recent years, the emergence of azole-resistant *Aspergillus* strains has substantially risen in Portugal, with the use of antifungal drugs per capita increasing more than in other European countries [48–50]. The release of over-the-counter antifungals and the increased industry marketing have encouraged and promoted self-medication, enabling uncontrolled, inadequate, and prolonged azole utilization. Moreover, azoles are also widely used in the agriculture and wood industry, contributing to prolonged exposure and resistance development [51–53]. To tackle this concern and prevent therapeutic failures, it is crucial to prioritize antifungal susceptibility testing of clinical isolates. This practice ensures the accurate identification of fungal species to tailor antifungal treatment strategies, avoiding the emergence of resistant strains. Despite Portugal being well-prepared in comparison to other European countries, this is still an area requiring improvement, as susceptibility testing should be performed routinely and include both yeast and mold-directed tests. A straightforward approach to help mitigate this challenge could involve, for instance, the use of multiplex real-time PCR assays, which would allow for the direct differentiation between susceptible and resistant strains.

According to the WHO, the most essential systemic antifungals encompass amphotericin B, in deoxycholate and liposomal formulations, anidulafungin, caspofungin, fluconazole, flucytosine, itraconazole, micafungin, and voriconazole [54]. Amphotericin B deoxycholate and lipid complex, anidulafungin, and flucytosine presented the lowest availability among Portuguese institutions. These numbers might be explained in part by the high anidulafungin costs when compared to other interchangeable echinocandins and the difficulties in acquiring amphotericin B deoxycholate and flucytosine in Portugal [55]. It is worth noting, however, that not all participating institutions have the ability to administer antifungal treatment, as they do not operate as treating centers. Despite the remaining drugs presenting better availability, Portugal still falls behind the European average for median-income countries in terms of access to echinocandins and triazoles, suggesting it might not be adequately prepared compared to counterparts with similar economic profiles. For instance, despite *Candida* spp. being perceived as the most significant fungal pathogen, echinocandins are the least frequently used antifungal. Our study thus highlights the need to improve the accessibility and distribution of antifungal drugs in Portugal to align with the country's specific fungal pathogen profile.

Portugal is still very much behind concerning TDM, with only a few institutions with the capacity to conduct such analyses. This process holds significant importance as it provides insight into appropriate drug dosages and potential adverse effects, enabling treatment optimizations [56]. Besides flucytosine, only the prescription of certain triazoles is monitored in these institutions. This represents a critical limitation since not all the antifungal classes are being covered, with a specific focus on echinocandins, which should be of special interest due to the high incidence of invasive *Candida* infections. This situation is particularly worrisome as most of the limited TDM procedures conducted are outsourced, and given the often inability to resort to the same provider this follow-up service.

Our study has several limitations that should be acknowledged. The available information and data do not include all the institutions that manage IFD in Portugal. Also important is the lack of information concerning the turnaround time of the available tests, particularly when outsourced, as this could impact the clinical effectiveness of such tests. The absence of data regarding the outsourcing of critical techniques, namely DNA sequencing and antifungal susceptibility testing, is also a limitation. Finally, data collection for this survey took place during the COVID-19 pandemic. This context may have imposed time restrictions and increased workload on laboratory professionals, microbiologists, and infectious disease specialists, potentially affecting the accuracy of the survey. Furthermore, the data obtained from institutions with greater experience and capacity in diagnosing and treating IFD may not fully represent smaller institutions. Variations in resources, expertise, and infrastructure across different types of healthcare facilities can significantly impact the management of these infections.

In summary, Portugal is well-prepared to manage IFD, but there are limitations to be addressed. These include ensuring widespread access to specific diagnostic tools, eliminating the high rates of outsourcing, improving the availability and suitability of antifungal drugs, and prioritizing the implementation of TDM across institutions. Addressing these limitations is crucial for facilitating earlier diagnosis and effective treatment of patients, ultimately improving outcomes in the management of IFD in Portugal.
